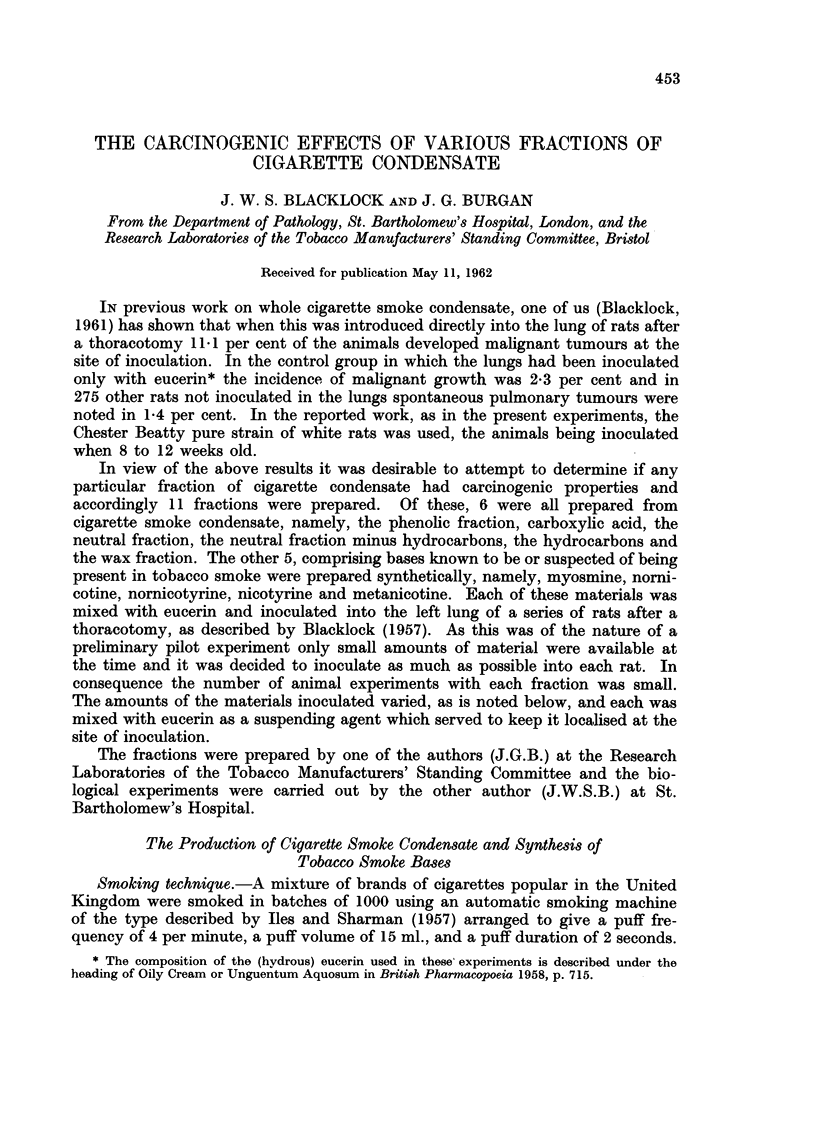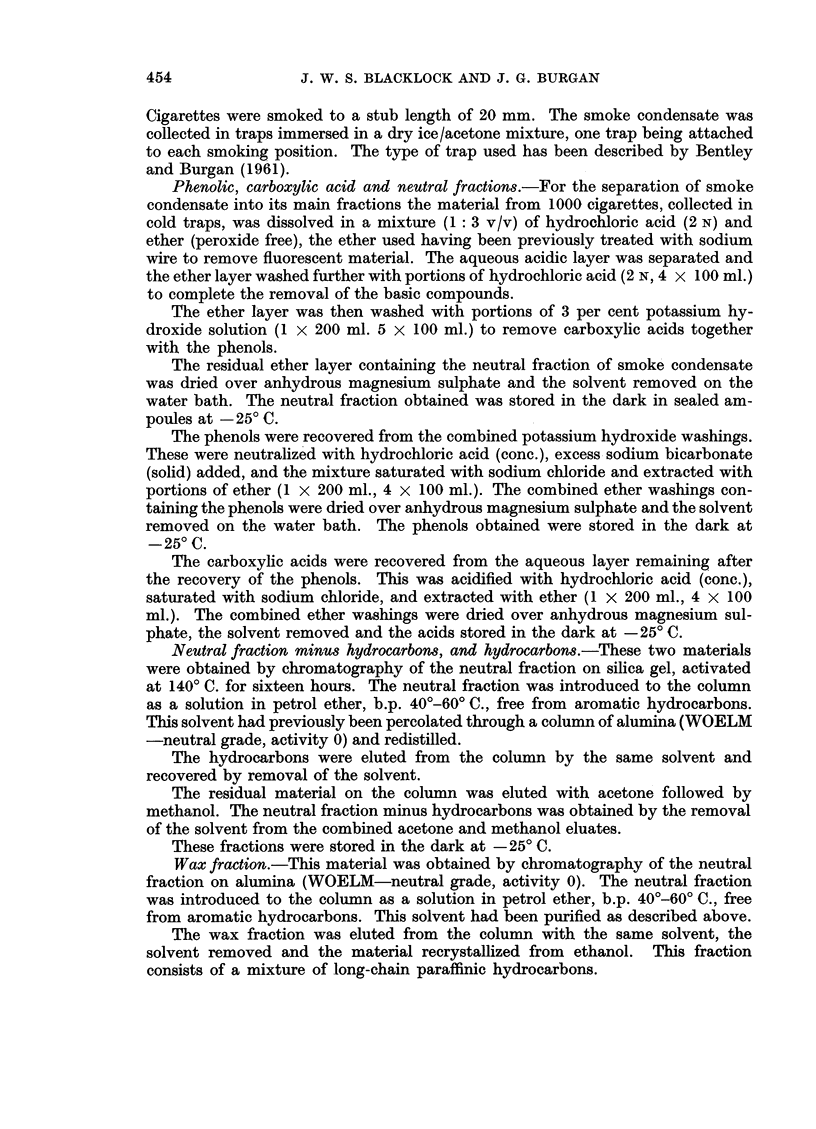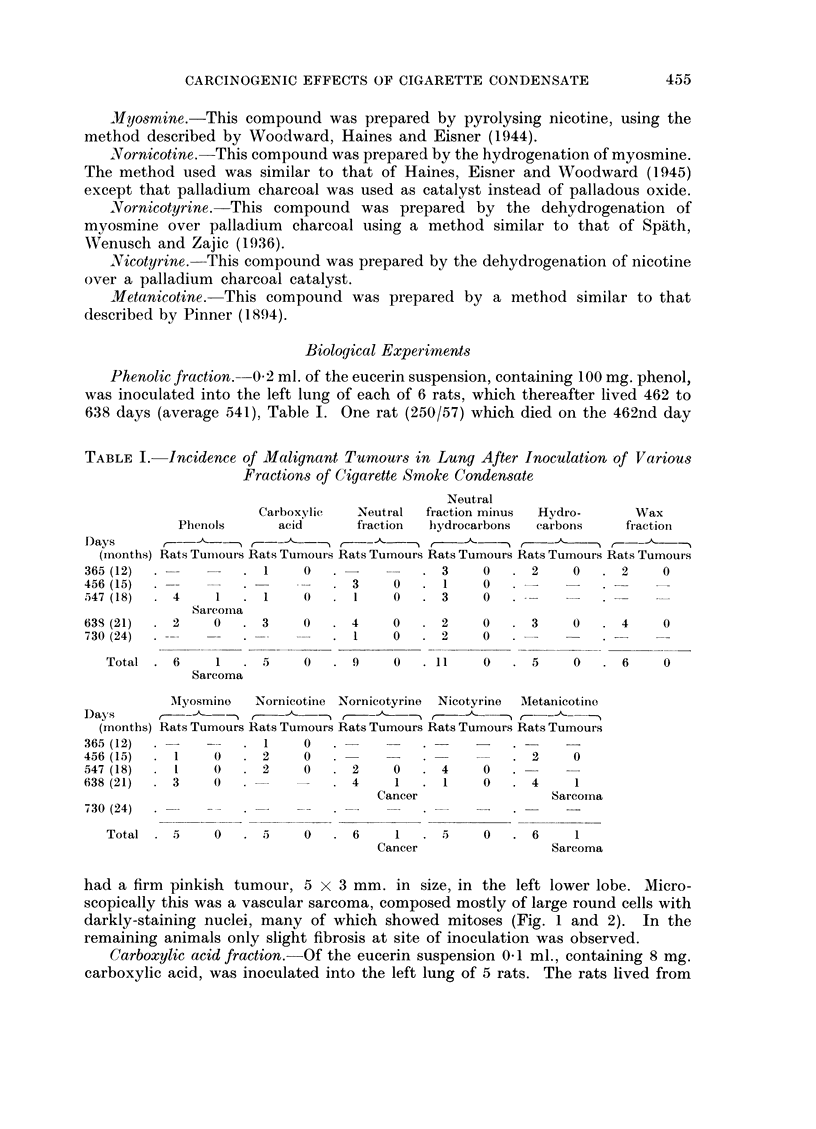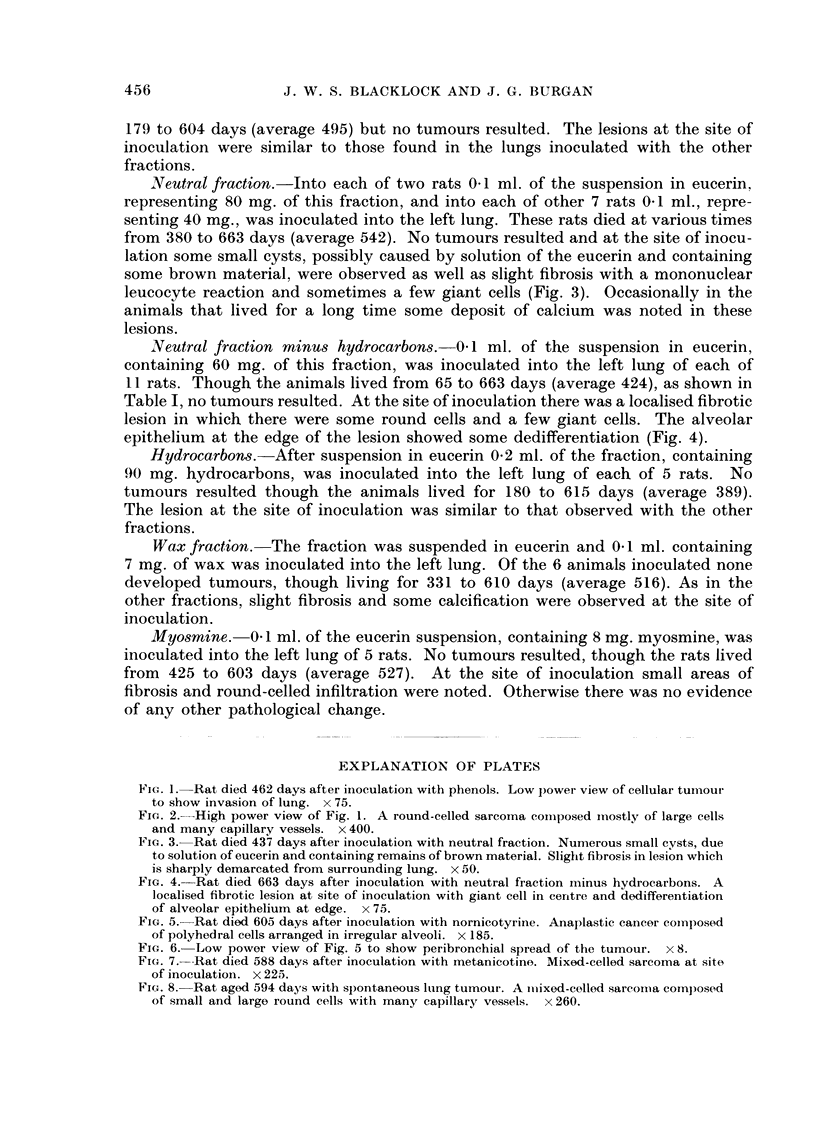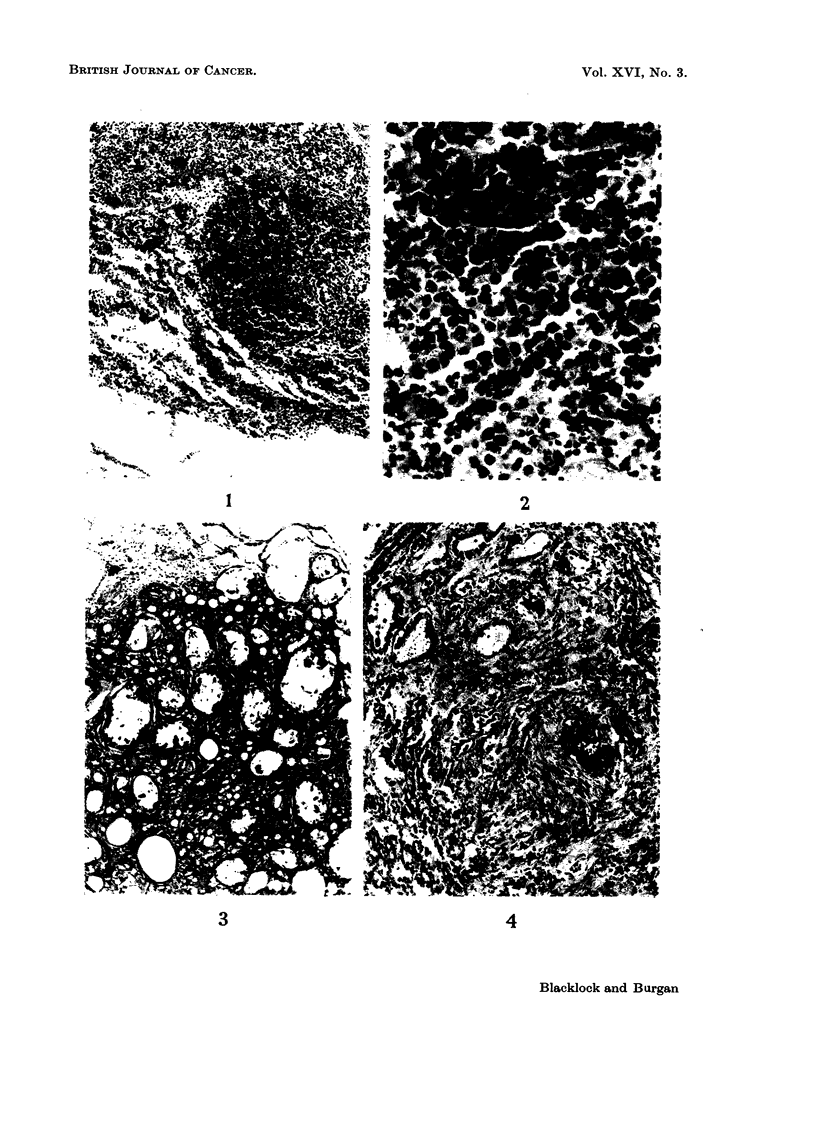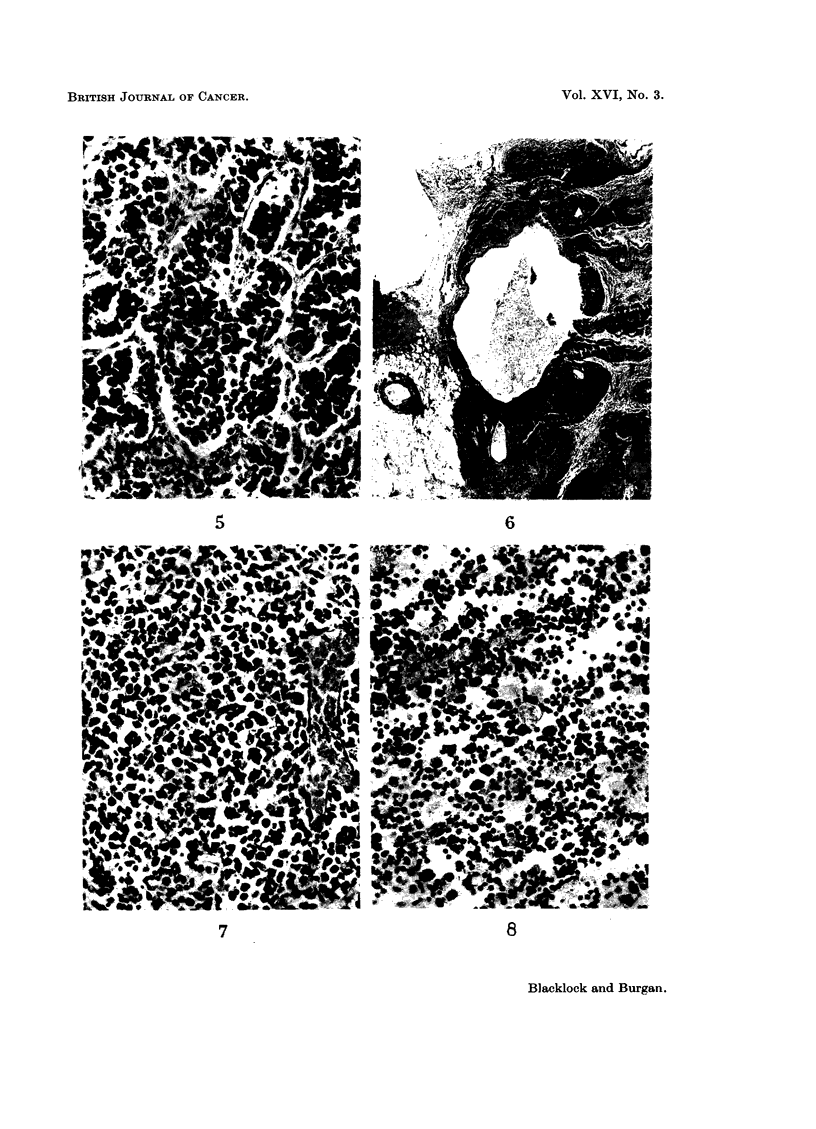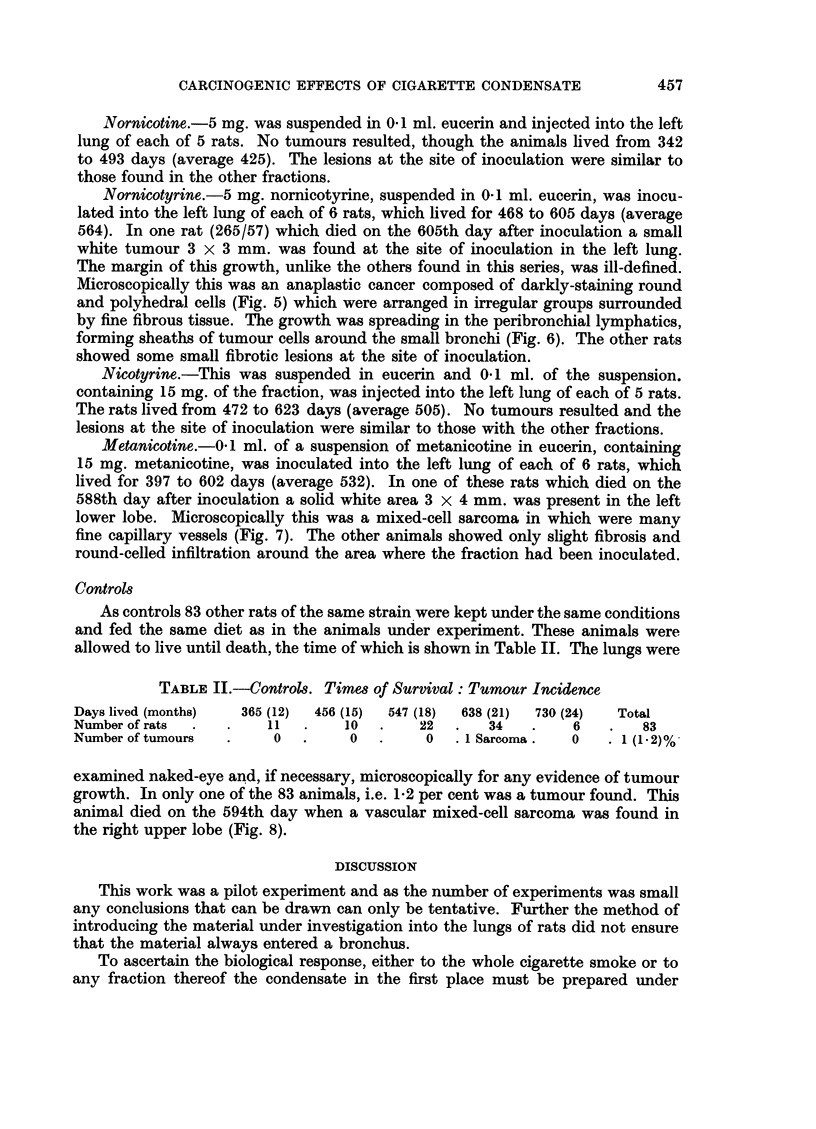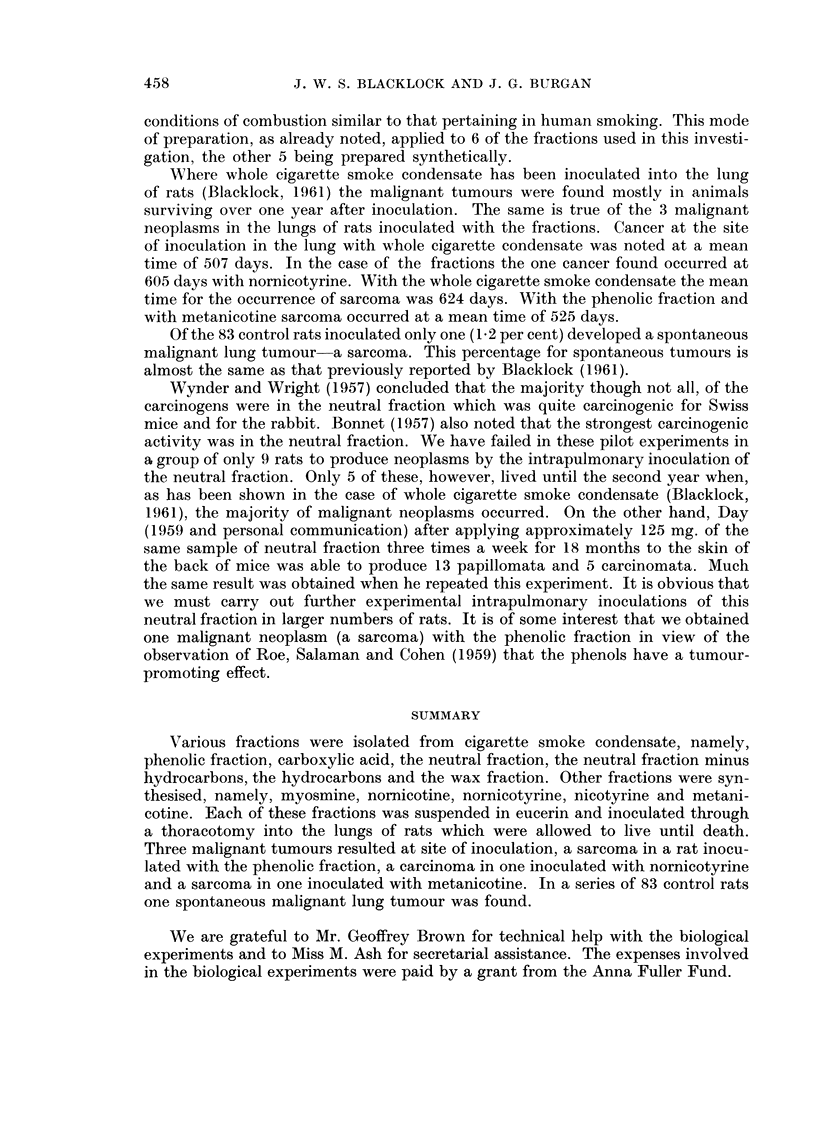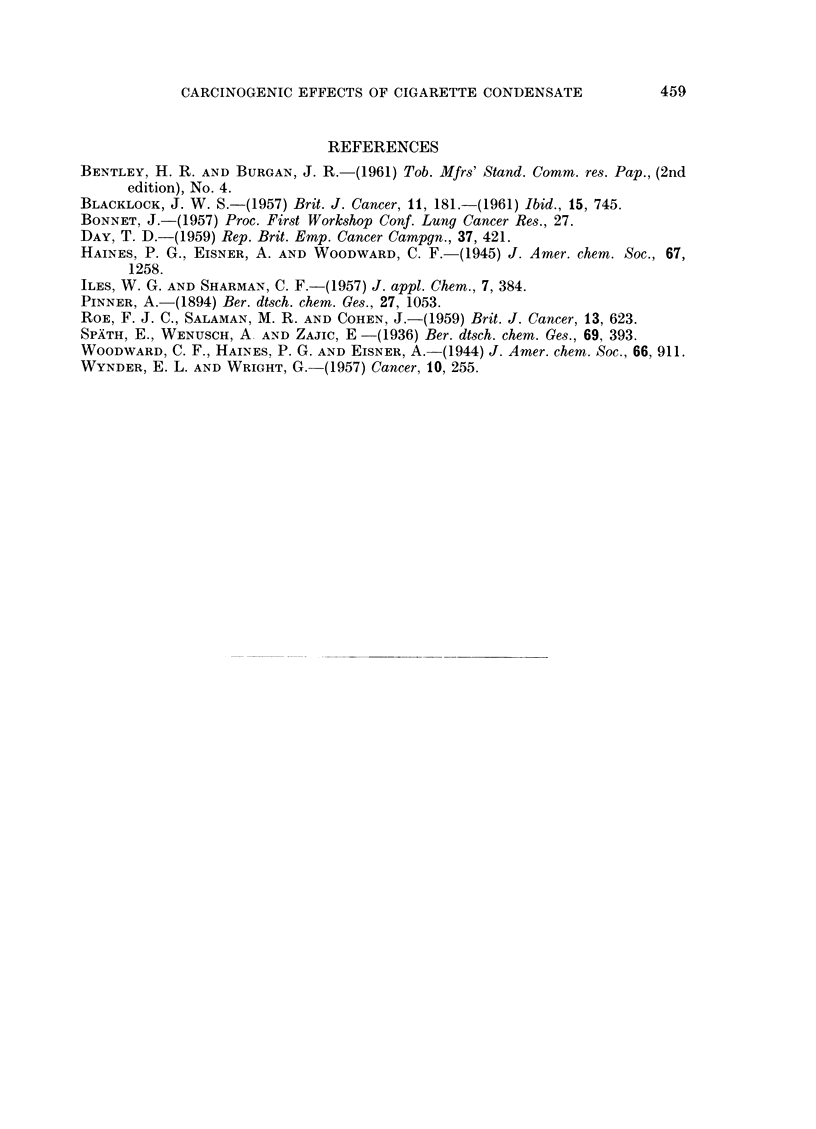# The Carcinogenic Effects of Various Fractions of Cigarette Condensate

**DOI:** 10.1038/bjc.1962.50

**Published:** 1962-09

**Authors:** J. W. S. Blacklock, J. G. Burgan

## Abstract

**Images:**


### THE CARCINOGENIC EFFECTS OF VARIOUS FRACTIONS OF CIGARETTE CONDENSATE

J. W. S. BLACKLOCK AND J. G. BURGAN

*From the Department of Pathology, St. Bartholomew's Hospital, London, and the Research Laboratories of the Tobacco Manufacturers' Standing Committee, Bristol*

Received for publication May 11, 1962

IN previous work on whole cigarette smoke condensate, one of us (Blacklock, 1961) has shown that when this was introduced directly into the lung of rats after a thoracotomy 11·1 per cent of the animals developed malignant tumours at the site of inoculation. In the control group in which the lungs had been inoculated only with eucerin* the incidence of malignant growth was 2·3 per cent and in 275 other rats not inoculated in the lungs spontaneous pulmonary tumours were noted in 1·4 per cent. In the reported work, as in the present experiments, the Chester Beatty pure strain of white rats was used, the animals being inoculated when 8 to 12 weeks old.

In view of the above results it was desirable to attempt to determine if any particular fraction of cigarette condensate had carcinogenic properties and accordingly 11 fractions were prepared. Of these, 6 were all prepared from cigarette smoke condensate, namely, the phenolic fraction, carboxylic acid, the neutral fraction, the neutral fraction minus hydrocarbons, the hydrocarbons and the wax fraction. The other 5, comprising bases known to be or suspected of being present in tobacco smoke were prepared synthetically, namely, myosmine, nornicotine, nornicotyrine, nicotyrine and metanicotine. Each of these materials was mixed with eucerin and inoculated into the left lung of a series of rats after a thoracotomy, as described by Blacklock (1957). As this was of the nature of a preliminary pilot experiment only small amounts of material were available at the time and it was decided to inoculate as much as possible into each rat. In consequence the number of animal experiments with each fraction was small. The amounts of the materials inoculated varied, as is noted below, and each was mixed with eucerin as a suspending agent which served to keep it localised at the site of inoculation.

The fractions were prepared by one of the authors (J.G.B.) at the Research Laboratories of the Tobacco Manufacturers' Standing Committee and the biological experiments were carried out by the other author (J.W.S.B.) at St. Bartholomew's Hospital.

### *The Production of Cigarette Smoke Condensate and Synthesis of Tobacco Smoke Bases*

*Smoking technique.*—A mixture of brands of cigarettes popular in the United Kingdom were smoked in batches of 1000 using an automatic smoking machine of the type described by Iles and Sharman (1957) arranged to give a puff frequency of 4 per minute, a puff volume of 15 ml., and a puff duration of 2 seconds.

* The composition of the (hydrous) eucerin used in these experiments is described under the heading of Oily Cream or Unguentum Aquosum in *British Pharmacopoeia* 1958, p. 715.

Cigarettes were smoked to a stub length of 20 mm. The smoke condensate was collected in traps immersed in a dry ice/acetone mixture, one trap being attached to each smoking position. The type of trap used has been described by Bentley and Burgan (1961).

*Phenolic, carboxylic acid and neutral fractions.*—For the separation of smoke condensate into its main fractions the material from 1000 cigarettes, collected in cold traps, was dissolved in a mixture (1 : 3 v/v) of hydrochloric acid (2 N) and ether (peroxide free), the ether used having been previously treated with sodium wire to remove fluorescent material. The aqueous acidic layer was separated and the ether layer washed further with portions of hydrochloric acid (2 N, 4 × 100 ml.) to complete the removal of the basic compounds.

The ether layer was then washed with portions of 3 per cent potassium hydroxide solution (1 × 200 ml. 5 × 100 ml.) to remove carboxylic acids together with the phenols.

The residual ether layer containing the neutral fraction of smoke condensate was dried over anhydrous magnesium sulphate and the solvent removed on the water bath. The neutral fraction obtained was stored in the dark in sealed ampoules at −25° C.

The phenols were recovered from the combined potassium hydroxide washings. These were neutralized with hydrochloric acid (conc.), excess sodium bicarbonate (solid) added, and the mixture saturated with sodium chloride and extracted with portions of ether (1 × 200 ml., 4 × 100 ml.). The combined ether washings containing the phenols were dried over anhydrous magnesium sulphate and the solvent removed on the water bath. The phenols obtained were stored in the dark at −25° C.

The carboxylic acids were recovered from the aqueous layer remaining after the recovery of the phenols. This was acidified with hydrochloric acid (conc.), saturated with sodium chloride, and extracted with ether (1 × 200 ml., 4 × 100 ml.). The combined ether washings were dried over anhydrous magnesium sulphate, the solvent removed and the acids stored in the dark at −25° C.

*Neutral fraction minus hydrocarbons, and hydrocarbons.*—These two materials were obtained by chromatography of the neutral fraction on silica gel, activated at 140° C. for sixteen hours. The neutral fraction was introduced to the column as a solution in petrol ether, b.p. 40°–60° C., free from aromatic hydrocarbons. This solvent had previously been percolated through a column of alumina (WOELM—neutral grade, activity 0) and redistilled.

The hydrocarbons were eluted from the column by the same solvent and recovered by removal of the solvent.

The residual material on the column was eluted with acetone followed by methanol. The neutral fraction minus hydrocarbons was obtained by the removal of the solvent from the combined acetone and methanol eluates.

These fractions were stored in the dark at −25° C.

*Wax fraction.*—This material was obtained by chromatography of the neutral fraction on alumina (WOELM—neutral grade, activity 0). The neutral fraction was introduced to the column as a solution in petrol ether, b.p. 40°–60° C., free from aromatic hydrocarbons. This solvent had been purified as described above.

The wax fraction was eluted from the column with the same solvent, the solvent removed and the material recrystallized from ethanol. This fraction consists of a mixture of long-chain paraffinic hydrocarbons.

*Myosmine.*—This compound was prepared by pyrolysing nicotine, using the method described by Woodward, Haines and Eisner (1944).

*Nornicotine.*—This compound was prepared by the hydrogenation of myosmine. The method used was similar to that of Haines, Eisner and Woodward (1945) except that palladium charcoal was used as catalyst instead of palladous oxide.

*Nornicotyrine.*—This compound was prepared by the dehydrogenation of myosmine over palladium charcoal using a method similar to that of Späth, Wenusch and Zajic (1936).

*Nicotyrine.*—This compound was prepared by the dehydrogenation of nicotine over a palladium charcoal catalyst.

*Metanicotine.*—This compound was prepared by a method similar to that described by Pinner (1894).

### *Biological Experiments*

*Phenolic fraction.*—0·2 ml. of the eucerin suspension, containing 100 mg. phenol, was inoculated into the left lung of each of 6 rats, which thereafter lived 462 to 638 days (average 541), Table I. One rat (250/57) which died on the 462nd day had a firm pinkish tumour, 5 × 3 mm. in size, in the left lower lobe. Microscopically this was a vascular sarcoma, composed mostly of large round cells with darkly-staining nuclei, many of which showed mitoses (Fig. 1 and 2). In the remaining animals only slight fibrosis at site of inoculation was observed.

TABLE I.—*Incidence of Malignant Tumours in Lung After Inoculation of Various Fractions of Cigarette Smoke Condensate*

| Days | Phenols | | Carboxylic acid | | Neutral fraction | | Neutral fraction minus hydrocarbons | | Hydrocarbons | | Wax fraction | |
|---|---|---|---|---|---|---|---|---|---|---|---|---|
| (months) | Rats | Tumours | Rats | Tumours | Rats | Tumours | Rats | Tumours | Rats | Tumours | Rats | Tumours |
| 365 (12) | — | — | 1 | 0 | — | — | 3 | 0 | 2 | 0 | 2 | 0 |
| 456 (15) | — | — | — | — | 3 | 0 | 1 | 0 | — | — | — | — |
| 547 (18) | 4 | 1 Sarcoma | 1 | 0 | 1 | 0 | 3 | 0 | — | — | — | — |
| 638 (21) | 2 | 0 | 3 | 0 | 4 | 0 | 2 | 0 | 3 | 0 | 4 | 0 |
| 730 (24) | — | — | — | — | 1 | 0 | 2 | 0 | — | — | — | — |
| Total | 6 | 1 Sarcoma | 5 | 0 | 9 | 0 | 11 | 0 | 5 | 0 | 6 | 0 |

| Days | Myosmine | | Nornicotine | | Nornicotyrine | | Nicotyrine | | Metanicotine | |
|---|---|---|---|---|---|---|---|---|---|---|
| (months) | Rats | Tumours | Rats | Tumours | Rats | Tumours | Rats | Tumours | Rats | Tumours |
| 365 (12) | — | — | 1 | 0 | — | — | — | — | — | — |
| 456 (15) | 1 | 0 | 2 | 0 | — | — | — | — | 2 | 0 |
| 547 (18) | 1 | 0 | 2 | 0 | 2 | 0 | 4 | 0 | — | — |
| 638 (21) | 3 | 0 | — | — | 4 | 1 Cancer | 1 | 0 | 4 | 1 Sarcoma |
| 730 (24) | — | — | — | — | — | — | — | — | — | — |
| Total | 5 | 0 | 5 | 0 | 6 | 1 Cancer | 5 | 0 | 6 | 1 Sarcoma |

*Carboxylic acid fraction.*—Of the eucerin suspension 0·1 ml., containing 8 mg. carboxylic acid, was inoculated into the left lung of 5 rats. The rats lived from

179 to 604 days (average 495) but no tumours resulted. The lesions at the site of inoculation were similar to those found in the lungs inoculated with the other fractions.

*Neutral fraction.*—Into each of two rats 0·1 ml. of the suspension in eucerin, representing 80 mg. of this fraction, and into each of other 7 rats 0·1 ml., representing 40 mg., was inoculated into the left lung. These rats died at various times from 380 to 663 days (average 542). No tumours resulted and at the site of inoculation some small cysts, possibly caused by solution of the eucerin and containing some brown material, were observed as well as slight fibrosis with a mononuclear leucocyte reaction and sometimes a few giant cells (Fig. 3). Occasionally in the animals that lived for a long time some deposit of calcium was noted in these lesions.

*Neutral fraction minus hydrocarbons.*—0·1 ml. of the suspension in eucerin, containing 60 mg. of this fraction, was inoculated into the left lung of each of 11 rats. Though the animals lived from 65 to 663 days (average 424), as shown in Table I, no tumours resulted. At the site of inoculation there was a localised fibrotic lesion in which there were some round cells and a few giant cells. The alveolar epithelium at the edge of the lesion showed some dedifferentiation (Fig. 4).

*Hydrocarbons.*—After suspension in eucerin 0·2 ml. of the fraction, containing 90 mg. hydrocarbons, was inoculated into the left lung of each of 5 rats. No tumours resulted though the animals lived for 180 to 615 days (average 389). The lesion at the site of inoculation was similar to that observed with the other fractions.

*Wax fraction.*—The fraction was suspended in eucerin and 0·1 ml. containing 7 mg. of wax was inoculated into the left lung. Of the 6 animals inoculated none developed tumours, though living for 331 to 610 days (average 516). As in the other fractions, slight fibrosis and some calcification were observed at the site of inoculation.

*Myosmine.*—0·1 ml. of the eucerin suspension, containing 8 mg. myosmine, was inoculated into the left lung of 5 rats. No tumours resulted, though the rats lived from 425 to 603 days (average 527). At the site of inoculation small areas of fibrosis and round-celled infiltration were noted. Otherwise there was no evidence of any other pathological change.

### EXPLANATION OF PLATES

FIG. 1.—Rat died 462 days after inoculation with phenols. Low power view of cellular tumour to show invasion of lung. × 75.

FIG. 2.—High power view of Fig. 1. A round-celled sarcoma composed mostly of large cells and many capillary vessels. × 400.

FIG. 3.—Rat died 437 days after inoculation with neutral fraction. Numerous small cysts, due to solution of eucerin and containing remains of brown material. Slight fibrosis in lesion which is sharply demarcated from surrounding lung. × 50.

FIG. 4.—Rat died 663 days after inoculation with neutral fraction minus hydrocarbons. A localised fibrotic lesion at site of inoculation with giant cell in centre and dedifferentiation of alveolar epithelium at edge. × 75.

FIG. 5.—Rat died 605 days after inoculation with nornicotyrine. Anaplastic cancer composed of polyhedral cells arranged in irregular alveoli. × 185.

FIG. 6.—Low power view of Fig. 5 to show peribronchial spread of the tumour. × 8.

FIG. 7.—Rat died 588 days after inoculation with metanicotine. Mixed-celled sarcoma at site of inoculation. × 225.

FIG. 8.—Rat aged 594 days with spontaneous lung tumour. A mixed-celled sarcoma composed of small and large round cells with many capillary vessels. × 260.

1

2

3

4

Blacklock and Burgan

5

6

7

8

Blacklock and Burgan.

*Nornicotine.*—5 mg. was suspended in 0·1 ml. eucerin and injected into the left lung of each of 5 rats. No tumours resulted, though the animals lived from 342 to 493 days (average 425). The lesions at the site of inoculation were similar to those found in the other fractions.

*Nornicotyrine.*—5 mg. nornicotyrine, suspended in 0·1 ml. eucerin, was inoculated into the left lung of each of 6 rats, which lived for 468 to 605 days (average 564). In one rat (265/57) which died on the 605th day after inoculation a small white tumour 3 × 3 mm. was found at the site of inoculation in the left lung. The margin of this growth, unlike the others found in this series, was ill-defined. Microscopically this was an anaplastic cancer composed of darkly-staining round and polyhedral cells (Fig. 5) which were arranged in irregular groups surrounded by fine fibrous tissue. The growth was spreading in the peribronchial lymphatics, forming sheaths of tumour cells around the small bronchi (Fig. 6). The other rats showed some small fibrotic lesions at the site of inoculation.

*Nicotyrine.*—This was suspended in eucerin and 0·1 ml. of the suspension. containing 15 mg. of the fraction, was injected into the left lung of each of 5 rats. The rats lived from 472 to 623 days (average 505). No tumours resulted and the lesions at the site of inoculation were similar to those with the other fractions.

*Metanicotine.*—0·1 ml. of a suspension of metanicotine in eucerin, containing 15 mg. metanicotine, was inoculated into the left lung of each of 6 rats, which lived for 397 to 602 days (average 532). In one of these rats which died on the 588th day after inoculation a solid white area 3 × 4 mm. was present in the left lower lobe. Microscopically this was a mixed-cell sarcoma in which were many fine capillary vessels (Fig. 7). The other animals showed only slight fibrosis and round-celled infiltration around the area where the fraction had been inoculated.

*Controls*

As controls 83 other rats of the same strain were kept under the same conditions and fed the same diet as in the animals under experiment. These animals were allowed to live until death, the time of which is shown in Table II. The lungs were

TABLE II.—*Controls. Times of Survival : Tumour Incidence*

| Days lived (months) | 365 (12) | 456 (15) | 547 (18) | 638 (21) | 730 (24) | Total |
|---|---|---|---|---|---|---|
| Number of rats | 11 | 10 | 22 | 34 | 6 | 83 |
| Number of tumours | 0 | 0 | 0 | 1 Sarcoma | 0 | 1 (1·2)% |

examined naked-eye and, if necessary, microscopically for any evidence of tumour growth. In only one of the 83 animals, i.e. 1·2 per cent was a tumour found. This animal died on the 594th day when a vascular mixed-cell sarcoma was found in the right upper lobe (Fig. 8).

### DISCUSSION

This work was a pilot experiment and as the number of experiments was small any conclusions that can be drawn can only be tentative. Further the method of introducing the material under investigation into the lungs of rats did not ensure that the material always entered a bronchus.

To ascertain the biological response, either to the whole cigarette smoke or to any fraction thereof the condensate in the first place must be prepared under conditions of combustion similar to that pertaining in human smoking. This mode of preparation, as already noted, applied to 6 of the fractions used in this investigation, the other 5 being prepared synthetically.

Where whole cigarette smoke condensate has been inoculated into the lung of rats (Blacklock, 1961) the malignant tumours were found mostly in animals surviving over one year after inoculation. The same is true of the 3 malignant neoplasms in the lungs of rats inoculated with the fractions. Cancer at the site of inoculation in the lung with whole cigarette condensate was noted at a mean time of 507 days. In the case of the fractions the one cancer found occurred at 605 days with nornicotyrine. With the whole cigarette smoke condensate the mean time for the occurrence of sarcoma was 624 days. With the phenolic fraction and with metanicotine sarcoma occurred at a mean time of 525 days.

Of the 83 control rats inoculated only one (1·2 per cent) developed a spontaneous malignant lung tumour—a sarcoma. This percentage for spontaneous tumours is almost the same as that previously reported by Blacklock (1961).

Wynder and Wright (1957) concluded that the majority though not all, of the carcinogens were in the neutral fraction which was quite carcinogenic for Swiss mice and for the rabbit. Bonnet (1957) also noted that the strongest carcinogenic activity was in the neutral fraction. We have failed in these pilot experiments in a group of only 9 rats to produce neoplasms by the intrapulmonary inoculation of the neutral fraction. Only 5 of these, however, lived until the second year when, as has been shown in the case of whole cigarette smoke condensate (Blacklock, 1961), the majority of malignant neoplasms occurred. On the other hand, Day (1959 and personal communication) after applying approximately 125 mg. of the same sample of neutral fraction three times a week for 18 months to the skin of the back of mice was able to produce 13 papillomata and 5 carcinomata. Much the same result was obtained when he repeated this experiment. It is obvious that we must carry out further experimental intrapulmonary inoculations of this neutral fraction in larger numbers of rats. It is of some interest that we obtained one malignant neoplasm (a sarcoma) with the phenolic fraction in view of the observation of Roe, Salaman and Cohen (1959) that the phenols have a tumour-promoting effect.

### SUMMARY

Various fractions were isolated from cigarette smoke condensate, namely, phenolic fraction, carboxylic acid, the neutral fraction, the neutral fraction minus hydrocarbons, the hydrocarbons and the wax fraction. Other fractions were synthesised, namely, myosmine, nornicotine, nornicotyrine, nicotyrine and metanicotine. Each of these fractions was suspended in eucerin and inoculated through a thoracotomy into the lungs of rats which were allowed to live until death. Three malignant tumours resulted at site of inoculation, a sarcoma in a rat inoculated with the phenolic fraction, a carcinoma in one inoculated with nornicotyrine and a sarcoma in one inoculated with metanicotine. In a series of 83 control rats one spontaneous malignant lung tumour was found.

We are grateful to Mr. Geoffrey Brown for technical help with the biological experiments and to Miss M. Ash for secretarial assistance. The expenses involved in the biological experiments were paid by a grant from the Anna Fuller Fund.

### REFERENCES

BENTLEY, H. R. AND BURGAN, J. R.—(1961) *Tob. Mfrs' Stand. Comm. res. Pap.*, (2nd edition), No. 4.

BLACKLOCK, J. W. S.—(1957) *Brit. J. Cancer*, **11**, 181.—(1961) *Ibid.*, **15**, 745.

BONNET, J.—(1957) *Proc. First Workshop Conf. Lung Cancer Res.*, 27.

DAY, T. D.—(1959) *Rep. Brit. Emp. Cancer Campgn.*, **37**, 421.

HAINES, P. G., EISNER, A. AND WOODWARD, C. F.—(1945) *J. Amer. chem. Soc.*, **67**, 1258.

ILES, W. G. AND SHARMAN, C. F.—(1957) *J. appl. Chem.*, **7**, 384.

PINNER, A.—(1894) *Ber. dtsch. chem. Ges.*, **27**, 1053.

ROE, F. J. C., SALAMAN, M. R. AND COHEN, J.—(1959) *Brit. J. Cancer*, **13**, 623.

SPÄTH, E., WENUSCH, A AND ZAJIC, E —(1936) *Ber. dtsch. chem. Ges.*, **69**, 393.

WOODWARD, C. F., HAINES, P. G. AND EISNER, A.—(1944) *J. Amer. chem. Soc.*, **66**, 911.

WYNDER, E. L. AND WRIGHT, G.—(1957) *Cancer*, **10**, 255.